# Neutralization of acyl CoA binding protein (ACBP) for the experimental treatment of osteoarthritis

**DOI:** 10.1038/s41418-025-01474-y

**Published:** 2025-03-13

**Authors:** Uxía Nogueira-Recalde, Flavia Lambertucci, Léa Montégut, Omar Motiño, Hui Chen, Sylvie Lachkar, Gerasimos Anagnostopoulos, Gautier Stoll, Sijing Li, Vincent Carbonier, Ester Saavedra Díaz, Francisco J. Blanco, Geert van Tetering, Mark de Boer, Maria Chiara Maiuri, Beatriz Caramés, Isabelle Martins, Guido Kroemer

**Affiliations:** 1 Centre de Recherche des Cordeliers, Equipe Labellisée par la Ligue Contre le Cancer, Université de Paris Cité, Sorbonne Université, Paris, France; 2 Metabolomics and Cell Biology Platforms, Gustave Roussy Cancer Center, Villejuif, France; 3 Unidad de Biología del Cartílago, Grupo de Investigación en Reumatología (GIR), Instituto de Investigación Biomédica de A Coruña (INIBIC), Complejo Hospitalario Universitario de A Coruña (CHUAC), Sergas, Universidad de A Coruña (UDC), A Coruña, Spain; 4 Unidad de Excelencia, Instituto de Biología y Genética Molecular (IBGM), Universidad de Valladolid – CSIC, Valladolid, Spain; 5 Departamento de Bioquímica y Biología Molecular, Fisiología, Genética e Inmunología, Instituto Universitario de Investigaciones Biomédicas y Sanitarias (IUIBS), Universidad de Las Palmas de Gran Canaria, Las Palmas de Gran Canaria, Spain; 6 Osasuna Therapeutics, Lausanne, Switzerland; 7 Department of Molecular Medicine and Medical Biotechnologies, University of Napoli Federico II, Napoli, Italy; 8 Department of Biology, Institut du Cancer Paris CARPEM, Hôpital Européen Georges Pompidou, AP-HP, Paris, France

**Keywords:** Rheumatic diseases, Autophagy

## Abstract

The plasma concentrations of acyl CoA binding protein (ACBP) encoded by the gene *diazepam binding inhibitor* (*DBI*) are increased in patients with severe osteoarthritis (OA). Here, we show that knee OA induces a surge in plasma ACBP/DBI in mice subjected to surgical destabilization of one hind limb. Knockout of the *Dbi* gene or intraperitoneal (i.p.) injection of a monoclonal antibody (mAb) neutralizing ACBP/DBI attenuates OA progression in this model, supporting a pathogenic role for ACBP/DBI in OA. Furthermore, anti-ACBP/DBI mAb was also effective against OA after its intraarticular (i.a.) injection, as monitored by sonography, revealing the capacity of ACBP/DBI to locally reduce knee inflammation over time. In addition, i.a. anti-ACBP/DBI mAb improved functional outcomes, as indicated by the reduced weight imbalance caused by OA. At the anatomopathological level, i.a. anti-ACBP/DBI mAb mitigated histological signs of joint destruction and synovial inflammation. Of note, i.a. anti-ACBP/DBI mAb blunted the OA-induced surge of plasma ACBP/DBI, as well as that of other inflammatory factors including interleukin-1α, interleukin-33, and tumor necrosis factor. These findings are potentially translatable to OA patients because joints from OA patients express both ACBP/DBI and its receptor GABA_A_Rγ2. Moreover, a novel mAb against ACBP/DBI recognizing an epitope conserved between human and mouse ACBP/DBI demonstrated similar efficacy in mitigating OA as an anti-mouse ACBP/DBI-only mAb. In conclusion, ACBP/DBI might constitute a promising therapeutic target for the treatment of OA.

## Introduction

Osteoarthritis (OA) is the most prevalent form of arthritis and a leading cause of disability among adults worldwide [1]. Characterized by the progressive destruction of articular cartilage, bone remodeling, osteophyte formation, synovial inflammation, degeneration of ligaments, menisci, and hypertrophy of the joint capsule [2, 3], OA primarily affects the knees, hips, hands, and spine [4, 5]. Invalidating OA currently affects 1 in 10 individuals over 60 years of age in the USA [6]. Since OA is painful and reduces mobility, it contributes to a vicious cycle in which OA-induced sedentary behavior locks aging patients in a state of progressive physical, mental, and social decline, ultimately culminating in loss of autonomy and frailty [7, 8]. Despite its high prevalence and substantial impact on quality of life, effective medical treatments for OA remain elusive, presenting a significant challenge for clinicians and researchers alike.

The pathophysiology of OA is multifactorial, involving a complex interplay of mechanical, biochemical, and genetic factors that contribute to the deterioration of joint structures and subsequent pain and dysfunction [9]. Mechanical stress due to obesity, joint malalignment, physical overactivity, occupational wear and tear, or previous injuries (such as ligament injuries, meniscal damage, and joint fractures), initiates and perpetuates the degradation of articular cartilage, which becomes increasingly prevalent with aging [10, 11]. Chondrocytes, the cells responsible for maintaining cartilage homeostasis, undergo phenotypic changes in OA, increasing the production of matrix-degrading enzymes such as matrix metalloproteinases and aggrecanases. Of note, chondrocyte cell death has been linked to an age-associated reduction in cytoprotective autophagy [12, 13]. Accordingly, cartilage-specific induction of autophagy by knockout of mTOR protects mice from experimental OA [14]. Similarly, induction of autophagy in chondrocytes by systemic administration of rapamycin [13], fenofibrate [15], or metformin [16] has been reported to alleviate OA in rodent models.

Subchondral bone remodeling also plays a crucial role in OA progression. Increased bone turnover and osteophyte formation alter joint biomechanics, contributing to further cartilage damage. Synovial inflammation, characterized by the infiltration of immune cells and elevated levels of pro-inflammatory cytokines like interleukin-1β (IL1β) and tumor necrosis factor-α (TNFα), exacerbates the disease by promoting additional catabolic activity and pain [17, 18]. Obesity, diabetes, and metabolic syndrome are associated with an increased risk of OA likely due to the inhibition of autophagy [19] and the promotion of systemic inflammation [20].

Despite the detailed understanding of OA pathogenesis, current therapeutic options are largely palliative and focus on symptom management rather than disease modification. Non-pharmacological treatments include moderate exercise, physiotherapy, as well as surgical joint replacement [21, 22]. Pharmacological treatments primarily include analgesics, non-steroidal anti-inflammatory drugs, and intraarticular corticosteroids [23]. While these medications can provide temporary relief from pain and inflammation, they do not halt or reverse the underlying joint degradation. Hence, OA constitutes a formidable clinical challenge due to its complex pathophysiology and the absence of effective disease-modifying treatments [24].

Acyl CoA binding protein (ACBP) encoded by diazepam binding inhibitor (DBI) is a tissue hormone that is released by multiple cell types into the extracellular space in response to stress. There it interacts with the γ2 subunit of the GABA receptor of the A type (protein symbol: GABRG2) to inhibit autophagy. This extracellular action of ACBP/DBI has been linked to several metabolic diseases, including obesity, diabetes, and liver pathologies such as steatohepatitis and fibrosis. Neutralization of ACBP/DBI, either through genetic modifications or by the use of specific antibodies, has been shown to induce autophagy and protect organs from damage caused by various stressors, such as ischemia and a variety of toxic agents [25–27]. ACBP/DBI plasma concentrations increase in several conditions that are OA risk factors including aging, diabetes, and obesity [28–30]. Plasma ACBP/DBI levels also correlate with signs of metabolic syndrome including hyperglycemia, hyperinsulinemia, hypercholesterinemia, elevated low-density lipoprotein, and reduced high-density lipoprotein [31]. In addition, ACBP/DBI correlates with general markers of inflammation including an elevation of the neutrophil/lymphocyte ratio and plasma levels of C reactive protein, interleukin-1β, and interleukin-6 [32, 33]. Of note, plasma ACBP/DBI concentrations exhibit an increase (by 23%) in patients (*n* = 1181) who underwent joint replacement due to severe knee or hip osteoarthritis in the forthcoming 2 years as compared to all other samples from the UK biobank (*n* = 49,754) [34]. Similarly, a meta-analysis of 72 distinct gene expression studies revealed that ACBP/DBI mRNA is significantly upregulated in the cartilage of patients with osteoarthritis as compared to healthy controls [35].

Recently, we developed methods to inhibit ACBP/DBI either genetically by a tamoxifen-inducible whole-body knockout of ACBP/DBI or pharmacologically by a neutralizing monoclonal antibody (mAb). Using these approaches, we could show that ACBP/DBI inhibition induces autophagy [28], which in turn protects various cell types (e.g., cardiomyocytes, hepatocytes, and pneumocytes) against cell death and reduces inflammation in several tissues (heart, liver, and lung) [26, 27, 31, 32]. Intrigued by these observations, we wondered whether ACBP/DBI might be involved in the pathogenesis of OA. Here, we show that systemic inhibition of ACBP/DBI by knockout or systemic injection of anti-ACBP/DBI mAb (α-DBI), as well as its local inhibition by intraarticular (i.a.) injection of α-DBI, exerts positive effects on OA.

## Materials and methods

### Cell culture

The immortalized human juvenile chondrocyte cell line, T/C28a2, was obtained as described previously [36]. This cell line was maintained in Dulbecco’s Modified Eagle Medium (DMEM, #11995-065, Gibco) supplemented with 10% Fetal Bovine Serum (FBS, 35-079-CV, Corning) and 100 U/mL Penicillin/100 µg/mL Streptomycin (P/S, #15070063, Gibco) at 37 °C and 5% CO_2_. Human SW982 synovial cells (American Type Culture Collection, ATCC®, HTB-93) were cultured in RPMI Medium 1640 (1X) (#11875-093, Gibco) supplemented with 10% FBS, 1% P/S, and 0.12 U/mL human insulin (#I0515, Sigma-Aldrich) and maintained at 37 °C in a humidified incubator containing 5% CO_2_. Both cell lines were seeded (T/C28a2, 1.5 × 10^5^ cells/well; SW982, 2 × 10^5^ cells/well) into 6-well plates (#353046, Corning) for 24 h. Chondrocytes were treated in DMEM + 2% FBS with IL6 (20 ng/mL, #130-095-365, Miltenyi), IL1β (5 ng/mL, #130-093-895, Miltenyi), and TNFα (10 ng/mL, #130-094-015) for 24 h. Synoviocytes were treated in RPMI + 2% FBS with the same pro-inflammatory human cytokines for 48 h.

### Immunodetection of proteins

Western blotting was performed using a chemiluminescence detection system. Cell lysates were prepared with 6 M urea/2% Sodium Dodecyl Sulfate (SDS) (#U6504, Sigma-Aldrich; #L4509, Sigma-Aldrich), and protein concentrations were determined using the Pierce™ BCA Protein Assay (#23225, Thermo Scientific). Protein extracts were boiled for 5 min at 95 °C in Laemmli sample buffer (#1610747, Bio-Rad). A total of 20 µg of protein from each cell lysate was separated on 4–20% SDS-polyacrylamide gels (#4561094, Bio-Rad) and transferred to PVDF membranes at a constant voltage of 2.5 A for 3 min using the Trans-Blot® Turbo™ Transfer System (#1704150, Bio-Rad). Non-specific binding sites were blocked by incubating the membranes for 1 h in 0.05% Tween 20 (#P1379, Sigma-Aldrich) (v/v in TBS) supplemented with 5% non-fat powdered milk (#A0830, PanReac AppliChem) (w/v in TBS). Membranes were then incubated overnight at 4 °C with primary antibodies specific for human ACBP/DBI (1:500, #Sc376853, Santa Cruz Biotechnology) or GABRG2 (1:1000, #ABIN754003, Antibodies Online). The GAPDH antibody (1:5000, #G9545, Sigma-Aldrich) was added for 1 h at room temperature (RT) as a loading control. Subsequently, membranes were incubated with horseradish peroxidase (HRP)-conjugated anti-rabbit IgG (1:5000, #NA934V, Sigma-Aldrich) or anti-mouse IgG (1:5000, #NA931V, Sigma-Aldrich) for 1 h at RT. After washing the membranes with TBS-T, protein bands were detected using a chemiluminescence substrate (#WBLUC0100, Millipore). Band intensity was quantified using Image Lab software (Bio-Rad) and expressed in relative units.

### Ethics approval and consent to participate

All methods were performed in accordance with the relevant guidelines and regulations, including the ARRIVE guidelines for animal studies. Human cartilage samples were obtained from the collection of samples for the investigation of Rheumatic Diseases, from Xerencia de Xestion Integrada de A Coruña and Instituto de Investigación Biomédica de A Coruña. This collection was registered in the National Registry of Biobanks, with registration code: C.0000424 and approved by the Ethics Committee of Galicia with registration code: 2013/107. Informed consent was obtained from all participants. No identifiable images of human participants are included in this study.

All animal experimentation procedures adhered to European and national institutional rules and guidelines (including the National Institutes of Health guide for the care and use of laboratory animals, NIH Publications No. 8023, revised 2011) and were approved by the local ethics committee (project numbers: #35912-202203151103707v4 and #31411-2021050411267667v3).

### Human cartilage samples

Articular cartilage explants from non-OA subjects (mean ± SD: 78.57 ± 18.42 years old, Mankin Score 0, *n* = 7), and OA subjects (mean ± SD: 84 ± 7.31 years old, Mankin Score 8, *n* = 5; mean ± SD: 77.75 ± 10.5 years old, Mankin Score 12, *n* = 4) were employed to quantify the expression of both ACBP/DBI and GABRG2 proteins. OA was diagnosed according to the Mankin Score [23, 37, 38].

### Immunohistochemistry

Human cartilage samples from non-OA and OA donors were fixed in 10% zinc-buffered formalin for 24 h. Knee joints from C57Bl/6J mice with OA induced by destabilization of the medial meniscus (DMM) + medial collateral ligament (MCL) were fixed in 4% buffered paraformaldehyde overnight at 4 °C and decalcified in TBD-2 for 48 h (REF #12607926 Thermo Fisher Scientific). Then, the two types of samples were embedded in paraffin, sliced to 4 μm thickness deparaffinized in xylene, and rehydrated in graded ethanol and water. For antigen unmasking, 10 mM sodium citrate (pH = 6.0) at 95 °C was added to the sections for 15 min and renewed every 3 min. Next, slides were washed with water for 15 min at RT and a Bloxall® pre-block solution (Vector Labs, #SP-6000) was added for 10 min. Afterward, samples were washed with 1% TBS-T and blocked with 10% goat serum for 1 h at RT (Vector Labs, #MP-7401). Then, sections were incubated with primary antibodies for human DBI (1:50, Santa Cruz #sc376853), mouse DBI (1:500, Abcam #Ab231910), human GABRG2 (1:200, Antibodies Online #ABIN754003) or mouse LC3 (1:750, MBL #PM036) overnight at 4 °C. After washing with 1% TBS-T, sections were incubated with HRP rabbit/mouse secondary antibody (ImmPRESS HRP kit, Vector labs #MP-7401) for 30 min at RT and washed with 1% TBS-T. The signal was developed with Diaminobenzidine (DAB)-Peroxidase substrate kit (Vector Labs, #SK-4100). Finally, sections were mounted with DePex (Sigma-Aldrich, #06522). Multiple images of each slide were taken using an Olympus BX61 microscope. The number of DBI, GABRG2, and LC3-positive cells was quantified using ImageJ (National Institute of Health, USA). Uncropped immunoblots are shown in supplementary data.

### Animal experimentation

Mice were housed in a temperature-controlled environment with 12 h light/dark cycles and were fed with diet and water *ad libitum*.

### Induction of ACBP/DBI knockout

Male C57Bl/6J mice bearing a floxed exon 2 of *Acbp/Dbi* gene in homozygosity as well as a ubiquitously expressed tamoxifen-inducible transgene coding for the *Cre* recombinase in homozygosity (genotype: *UBC- cre/ERT2*::*Acbp/Dbi*^f/f^, abbreviated as *Dbi*^−/−^) or “wild type” (WT) control mice (genotype: *Acbp/Dbi*^*f/f*^ without CRE) were injected with tamoxifen (i.p. 75 mg/KG BW tamoxifen/mouse daily during 5 days). Prior to injection, tamoxifen was diluted in corn oil (90%) + ethanol (10%) at a concentration of 20 mg/mL and shaken overnight at 37 °C as previously reported [28].

### Surgical induction of OA

Fifteen-weeks-old male C57BL/6JOlaHsd mice (Envigo, Gannat, France) were subjected to destabilization of the knee joint of the right posterior limb by cutting the MCL and surgical DMM as described [39, 40]. Sham procedures were performed by subjecting the control left knee to an operation involving only the opening of the joint capsule without ligament transection and meniscectomy.

### Treatment of OA with anti-ACBP/DBI antibody

Two weeks after surgical OA-inducing or sham procedures, mice were subjected to right intraarticular (i.a.) injections of anti-ACBP/DBI mAb (either clone 7G4a or clone 82B2G9) or isotype control (mouse IgG2a or IgG1, respectively) at concentrations of 1.25 ng/μL in PBS and an injection volume of 8 µL (i.e., 10 ng of antibody per joint) twice per week for 12 weeks. This dose was chosen after preliminary experiments involving small groups of mice. When mice were sacrificed, the knee articulations were excised and subjected to paraformaldehyde fixation for 24 h and subsequently decalcified for 48 h. In some experiments, 7G4a mAb and isotype control IgG2a were not injected intraarticularly but intraperitoneally (i.p.) at a dose of 5 mg/kg body weight, three times per week (as detailed in the legend).

### Functional assessment of OA

We used a static weight-bearing incapacitance test (Harvard Apparatus, Inc.—Panlab—Bioseb, Massachusetts, United States) to monitor the distribution of weight between the left and the right limbs in mice. In detail, mice were placed into a size-adjustable holder specially designed to naturally maintain the subjects positioned on two separate sensor plates. These sensors allowed the measurement of the weight distribution (in grams) in each hind paw reflecting spontaneous postural changes. Each measurement had a duration of 3 s and a total of 10 measurements were made per mouse. The recorded data were displayed in a unit that shows real-time weighing curves for left and right paws, as well as the static values. This unit assesses fast postural changes over the test period due to its 1000 Hz sampling frequency. In the absence of hind paw injury, animals apply equal weight to both hind paws, indicating a postural equilibrium. After unilateral hind paw tissue injury, a change in the weight distribution on the sensor occurs, depending on the level of discomfort [41]. These measurements were performed on a weekly basis by the same experimenter.

### Ultrasound biomicroscope B-mode analysis

An ultrasound biomicroscope (UBM) Vevo®3100 Imaging System, FUJIFILM Visualsonics, Toronto, Canada was employed to measure the surface area occupied by the tibia-femur triangle as well as hypoechographic zones, which both increase in OA, to determine UBM score, following published protocols [42, 43]. These measurements were performed on live anesthetized mice using 2% isoflurane in air, allowing for monthly examinations of the same mice. To monitor the development of the disease, each mouse was placed on a heated platform in a supine position and the hair was removed from the paws with depilatory cream. To perform the ultrasound examination the knee was placed at 90°. The high-frequency MX550D transducer (40 MHz) was used in a sagittal and transversal position to obtain optimal anatomical observation of the knee. Several images were acquired, using frame-based modes derived from B-mode data. After examining the joint, mice were deprived of isoflurane and a warm light was provided to facilitate awakening. To visualize morphological changes in the joint of this OA model, three ultrasound images of each knee were analyzed.

### Histological assessment of OA

Safranin O-Fast Green staining [44] on paraffin-embedded sagittal knee slices (thickness: 4 µm) was performed to determine the following parameters in the femoral condyle and in the tibial plateau: OARSI grade (which determines the severity of cartilage damage by attributing a score to erosion, destruction and calcification), OARSI stage (which determines the percentage of cartilage damage), as well as the composite OARSI score (grade multiplied by stage) [44, 45]. In addition, we determined the synovial inflammation score, which results as the numerical sum of three scores measuring (i) enlargement of the synovial lining layer, (ii) the reduction of the cellularity of synovial stroma with increase in multicellularity, as well as the later formation of pannus and rheumatoid granulomas, and (iii) the density of the synovial leukocyte infiltrate culminating in the formation of follicle-like aggregates [46].

### Biochemical characterization of monoclonal antibodies 7G4a and 82B2G9

The mAb 82B2G9 against the recombinant mouse ACBP/DBI protein was developed by ProtéoGenix (Schiltigheim, France) using hybridoma technology. This antibody was chosen based on its capability to detect the human and murine ACBP/DBI protein in both ELISA and Western blot assays. Furthermore, its specificity was validated through Western blot analyses using human and mouse ACBP/DBI knockout cell lines. Epitope mapping was conducted at Biosynth BV (Lelystad, The Netherlands) by linear and conformational epitope mapping. Linear peptides were synthesized based on the amino acid sequence of the target protein, using standard Fmoc-chemistry and deprotected using trifluoric acid with scavengers. For conformational mapping, the constrained peptides were synthesized on chemical scaffolds to reconstruct conformational epitopes, using Chemically Linked Peptides on Scaffolds (CLIPS) technology [47]. The binding of antibody to each peptide was tested in a PEPSCAN-based ELISA. The 82B2G9 antibody exhibited strong signals on both human and mouse sequences of ACBP/DBI, with similar putative epitopes identified. For the human ACBP/DBI protein sequence, the core epitope was determined as SPDEEMLFIYG, and for the mouse ACBP/DBI protein sequence, it was PTDEEMLFIYS. 7G4a mAb did not show strong binding to peptides derived from human ACBP/DBI but exhibited strong binding to some peptides derived from mouse ACBP/DBI protein. The core epitope in mouse ACBP/DBI recognized by 7G4a was identified as DRPGLLDL. For Western blot analyses, human and mouse recombinant ACBP/DBI protein were boiled for 5 min in Laemmli sample buffer, and 10 ng, 25 ng, 50 ng and 100 ng amounts of protein were separated on 4–12% Bis-Tris acrylamide precast gels (Thermo Fisher Scientific) and electro-transferred to nitrocellulose (Bio-Rad) membrane at a constant voltage of 100 V at 4 °C for 1.5 h. Unspecific binding sites of the membranes were saturated by incubating for 1 h in 0.05% Tween 20 (v:v in TBS) supplemented with 5% non-fat powdered milk (w:v in TBS). Subsequently, proteins were determined by overnight incubation of membranes with 82B2G9 antibody (1:500). Red ponceau was used to control equal loading of lanes. The blots were revealed using appropriate HRP-labeled secondary antibodies (Southern Biotech, AL, USA) plus ECL prime chemiluminescent substrate (Thermo Fisher Scientific). Different exposure times were utilized for each blot with a charged coupling device camera in a luminescent image analyzer LAS 4000 (GE Healthcare, IL, USA) to ensure the linearity of the band intensities. Quantification of proteins was carried out by densitometric analysis of the bands using ImageJ software (http://imagej.nih.gov) and was expressed as relative expression levels.

### Measurement of ACBP/DBI concentrations by ELISA

Mouse plasma was obtained from blood samples collected in lithium heparin tubes and centrifuged at 8500 rpm for 10 min at 4 °C. ACBP/DBI concentrations were measured using an ELISA assay as previously reported [31, 48]. Briefly, high-binding 96-well plates (Corning) were coated with 100 µL/well of anti-ACBP/DBI capture antibody (1 µg/mL, diluted in PBS) and incubated overnight at 4 °C. After washing, plates were blocked with 1% BSA in PBS-Tween 20 for 2 h at RT. Samples (murine plasma 1/20) and standards were added in 100 µL volumes and incubated for 2 h at RT. Plates were washed and incubated with 100 µL of the detection antibody (1 µg/mL) for 1 h at RT, followed by incubation with HRP-conjugated avidin (1/1000 for murine) for 30 min. After washing, 100 µL of TMB substrate was added and incubated in the dark for 10–30 min, followed by 50 µL of stop solution (2 M H_2_SO_4_). Absorbance was read at 450 nm using a FLUOstar OPTIMA microplate reader.

### Plasma cytokine multiplex analysis

Plasma cytokine concentrations were determined using a proximity extension assay with the Target 48 Mouse Cytokine panel (Olink, #93400) according to the manufacturer’s instructions. Briefly, 1 µL of plasma from fresh aliquots stored at −80 °C was thawed and incubated for 16 h at 4 °C in an incubation mix containing cytokine-specific antibody pairs, each coupled to forward and reverse probes. Extension of the complementary probes occurred on a SimpliAmp thermal cycler (Thermo Fisher, #A24811) and was possible only when both antibodies corresponding to a single cytokine were in close proximity, binding to neighboring epitopes on the target cytokine. For detection, an IFC 48.48 microfluidic chip (Olink, #93007) was primed and loaded with the samples and probes using an MX controller (Standard BioTools), and real-time PCR was performed on a BioMark HD system (Standard BioTools). PCR data analysis was conducted using the BioMark HD Real-Time PCR Analysis software (Standard BioTools), with automatic (global) Ct threshold determination set using the following parameters: quality threshold = 0.5, linear baseline correction. Data processing, quality control, and determination of absolute concentrations were performed using the Olink® NPX Signature software (v1.13.0).

The absolute concentrations were imported into R (version 4.3.3) and log2-fold change-transformed data were visualized using the ComplexHeatmap package (version 2.16.0). The distribution of log2-transformed values was tested for normality using the Shapiro–Wilk test. Cytokines that followed a normal distribution across the four groups were analyzed using a two-way analysis of variance (ANOVA), followed by Tukey’s HSD test for pairwise comparisons. Cytokines that did not follow a normal distribution were analyzed using the Kruskal–Wallis test, followed by Dunn’s post-hoc test, with Benjamini-Hochberg correction applied for multiple comparisons.

### Acute liver damage in mice

To induce hepatotoxicity, 12-week-old male C57BL/6 mice were treated intraperitoneally with 300 mg/kg acetaminophen (APAP, Sigma-Aldrich) for 16 h. Mice received intraperitoneal injections of anti-ACBP/DBI monoclonal antibodies (either clone 7G4a or clone 82B2G9) or their respective isotype control antibodies at a concentration of 2.5 μg/g body weight. The α-ACBP/DBI or IgG injections were administered 4 h before and immediately prior to the induction of hepatic injury. Elevations of transaminases, specifically aspartate transaminase (AST) and alanine transaminase (ALT), were measured in plasma using AST and ALT kits from Randox, following the manufacturer’s instructions. Alternatively, hepatic fibrosis was induced in mice through bile duct ligation, as previously described [26].

### Statistical analyses

For all figures, except where otherwise specified, a linear model was fitted using robust regression; *p* values were derived from the significance of model coefficients. For Supplementary Fig. 3, a robust post-hoc pairwise test was applied, directly generating *p* values. The data were analyzed within the R environment (version 4.2.1, citation: “R Core Team: A Language and Environment for Statistical Computing, R Foundation for Statistical Computing, Vienna, Austria. https://www.R-project.org/”), utilizing the “rlm” function from the MASS package (citation: “Venables WN, Ripley BD (2002). Modern Applied Statistics with S, Fourth edition. Springer, New York. ISBN 0-387-95457-0, https://www.stats.ox.ac.uk/pub/MASS4/”), the “rob.pval” function from the repmod package (citation: “https://CRAN.R-project.org/package=repmod”), and, for Supplementary Fig. 3, the “mcppb20” function from the WRS2 package (citation: “Mair P, Wilcox R (2020). Robust Statistical Methods in R Using the WRS2 Package. Behavior Research Methods, 52.”).

**Fig. 1:**
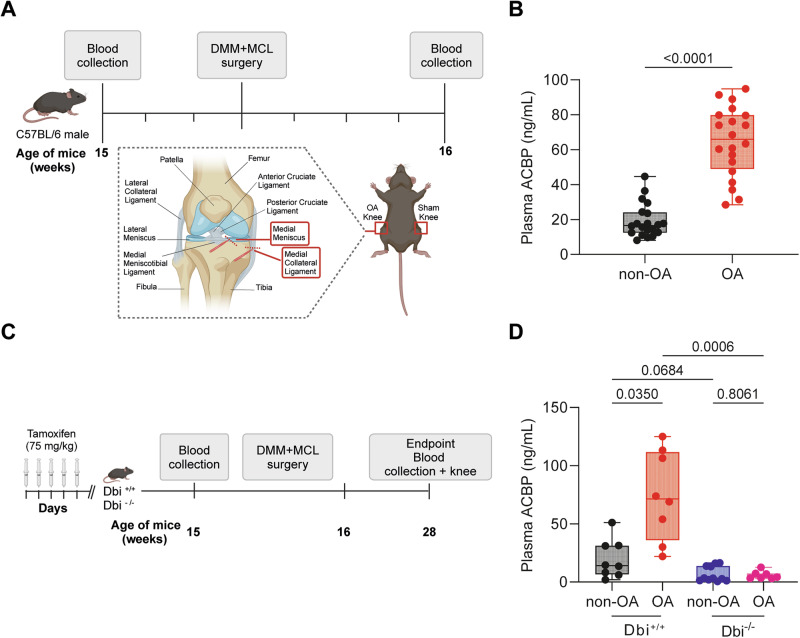
OA-induced increase in plasma ACBP/DBI levels in wild-type mice. **A** Schematic representation of the experimental design for osteoarthritis (OA) induction. OA was induced in 15-week-old male mice. Blood samples were collected both before and 1 week after OA induction to assess plasma ACBP/DBI levels. **B** Plasma ACBP/DBI results are displayed as box-and-whisker plots, with each dot representing an individual mouse (*n* = 20 mice per condition). For statistical analysis, *p* values were calculated using a Mann–Whitney test. **C** Schematic representation of the experimental design for osteoarthritis (OA) induction in ACBP/DBI-deficient mice. **D** Plasma ACBP/DBI results are displayed as box-and-whisker plots, with each dot representing an individual mouse (*n* = 8 to 11 mice per condition). For statistical analysis, *p* values were calculated using a Kruskal–Wallis test. Schematics in (**A**, **C**) were generated using BioRender.

**Fig. 2:**
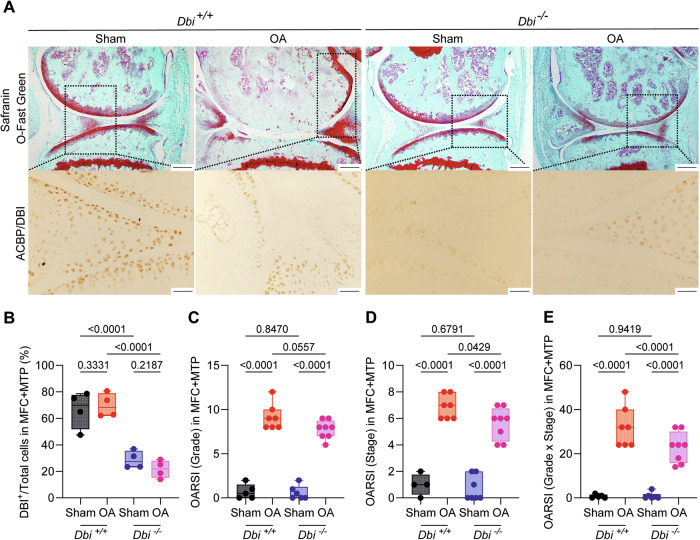
ACBP/DBI deficiency regulated cartilage degradation in osteoarthritis mice. **A** Representative Safranin O-Fast Green and ACBP/DBI-stained knee sections from wild-type and ACBP/DBI-deficient mice. Scale bar, 200 and 50 μm. **B** Quantification of ACBP/DBI-positive cells relative to total cells in the medial femoral condyle (MFC) and medial tibial plateau (MTP) was presented. Results are displayed as box-and-whisker plots, with each dot representing an individual mouse (*n* = 4). For statistical analysis, *p* values were calculated using two-way ANOVA corrected for multiple comparisons. **C** OARSI semiquantitative scoring for cartilage degradation (grade), **D** OARSI semiquantitative scoring for cartilage damage (stage), and **E** a combined index of grade and stage were used to evaluate the extent of cartilage damage. Results are displayed as box-and-whisker plots, with each dot representing an individual mouse (*n* = 4–8 mice per condition). For statistical analysis, *p* values were extracted from 2-way linear models, testing surgery significance within different genotypes (formula: Count ~ Genotype/Surgery) and testing genotype significance within surgery status (formula: Count ~ Surgery/Genotype).

For Figs. 1B, D, 2B and 7C, and Supplementary Fig. 4E, F, H, I, the Gaussian distribution of the results was assessed using the D’Agostino and Pearson normality test, the Shapiro–Wilk normality test, and the Kolmogorov-Smirnov test. Subsequently, an unpaired Student’s *t*-test or a two-way ANOVA was performed for pairwise or multiple comparisons, as indicated in the corresponding figures. Statistical analyses were conducted using GraphPad Prism 9 software. For the area under the curve (AUC) analysis, the “smplot2” a R package was used. We compare AUC values with a Wilcoxon test, a *p* value of <0.05 was considered statistically significant.

## Results

### OA causes a pathogenic upregulation of ACBP/DBI

Human OA is associated with an increase in ACBP/DBI plasma levels [34]. To establish a cause-effect relationship between these two phenomena, we measured ACBP/DBI levels in male C57BL/6 mice 3 days before and 4 days after OA-inducing surgical destabilization of the knee joint in the right hind limb, as well as in sham-operated control mice (Fig. 1A). In the OA model, the knee was surgically destabilized by transection of the MCL and DMM [39, 40] (Fig. 1A). Fifteen to 16 weeks after surgery, ACBP/DBI plasma concentrations were ~3 times higher in OA mice than in healthy controls (Fig. 1B). Then, we reproduced this approach in mice that can be subjected to tamoxifen-inducible whole-body knockout of floxed *Acbp/Dbi* (genotype: *UBC- cre/ERT2*::*Acbp/Dbi*^f/f^, abbreviated as *Dbi*^−/−^) or “wild type” (WT) control littermates (Acbp/Dbi^f/f^ without CRE). Mice of both genotypes were treated with tamoxifen, subjected to OA-inducing or sham surgery, and ACBP/DBI plasma levels were measured (Fig. 1C). As expected, the OA-induced surge in circulating ACBP/DBI was observed in WT mice but completely prevented in *Dbi*^−/−^ animals (Fig. 1D). Moreover, the percentage of cells staining positively for ACBP/DBI in joints were reduced in *Dbi*^−/−^ compared to WT mice (Fig. 2A, B), suggesting that the tamoxifen-induced knockout was also efficient in this location.

**Fig. 3:**
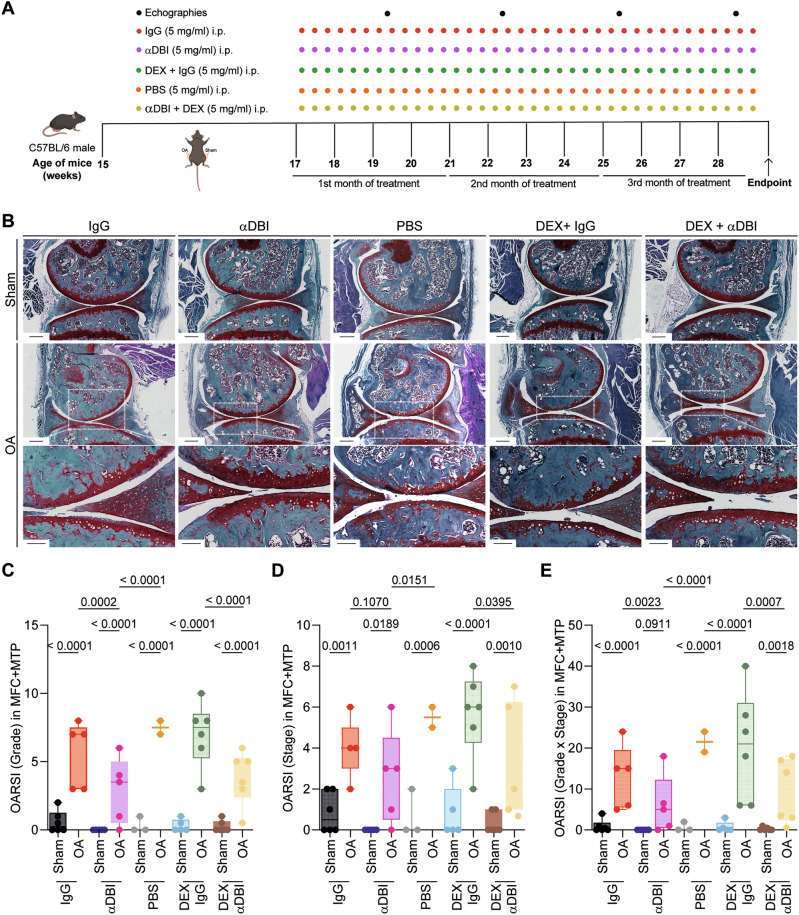
ACBP/DBI inhibition via monoclonal antibody treatment mitigated osteoarthritis severity. **A** Schematic representation of the experimental timeline. In 15-week-old male C57Bl/6 mice, mechanical destabilization of the right knee joint was induced via MCL/DMM surgery, while the left knee underwent sham surgery. After a 2-week post-surgical recovery, OA mice were intraperitoneally injected with either a monoclonal antibody (7G4a) that neutralizes extracellular ACBP/DBI (α-DBI) or an isotype control IgG antibody, administered thrice weekly. This treatment was optionally combined with the synthetic glucocorticoid dexamethasone (DEX). Ultrasound was performed every 3 weeks to monitor inflammation in the joint capsule. Schematic was generated using BioRender. **B** Representative Safranin O-Fast Green stained knee sections from different treatment groups, illustrating cartilage integrity are shown. Scale bars, 250 and 100 μm. **B**–**E** Histological evaluation of OA severity. **C** OARSI semiquantitative scoring for cartilage degradation (grade), **D** OARSI semiquantitative scoring for cartilage damage (stage), and **E** a combined index of grade and stage were used to evaluate the extent of cartilage damage. Results are displayed as box-and-whisker plots, with each dot representing an individual mouse (*n* = 2–6 mice per condition). For statistical analysis, *p* values were extracted from 2-way linear models, testing treatment significance within different genotypes (formula: Count ~ Genotype/Treatment) and testing genotype significance within different treatments (formula: Count ~ Treatment/Genotype).

When treated with the pro-inflammatory cytokines IL1β, interleukin-6 (IL6) or TNFα human T/C28a2 chondrocyte and SW982 synoviocyte cell lines upregulated ACBP/DBI and its receptor gamma-aminobutyric acid receptor subunit gamma-2 (GABRG2) at the protein level (Supplementary Fig. 1). Human OA specimens were also subjected to immunohistochemical detection of ACBP/DBI and GABRG2, indicating that both proteins, the ligand and the receptor, are present in the joints of OA patients (Supplementary Figs. 2 and 3). Of note, the knockout of ACBP/DBI in mice reduced the severity of OA, which was assessed on safranin O-Fast-stained tissue sections (Fig. 2A) according to Osteoarthritis Research Society International (OARSI) criteria [44, 45] measuring grade (Fig. 2C), stage (Fig. 2D) and their combination (i.e., grade multiplied by stage) as scores (Fig. 2E). As an alternative strategy of ACBP/DBI inhibition, we intraperitoneally injected OA mice with a mAb (7G4a) that neutralizes extracellular ACBP/DBI (abbreviated α-DBI) [28] or an isotype control IgG antibody. This treatment was optionally combined with the synthetic glucocorticoid dexamethasone (DEX) (Fig. 3A). While DEX alone did not inhibit OA severity, DBI neutralization alone or in combination with DEX did mitigate the histological signs of OA (Fig. 3B–E).

In conclusion, experimental OA is linked to the systemic upregulation of ACBP/DBI. In addition, knockout of ACBP/DBI or antibody-mediated neutralization of extracellular ACBP/DBI reduces the severity of OA, indicating a bidirectional crosstalk between OA and the ACBP/DBI system.

### Intraarticular injections of anti-ACBP/DBI antibody mitigate OA at the functional level

**Fig. 4:**
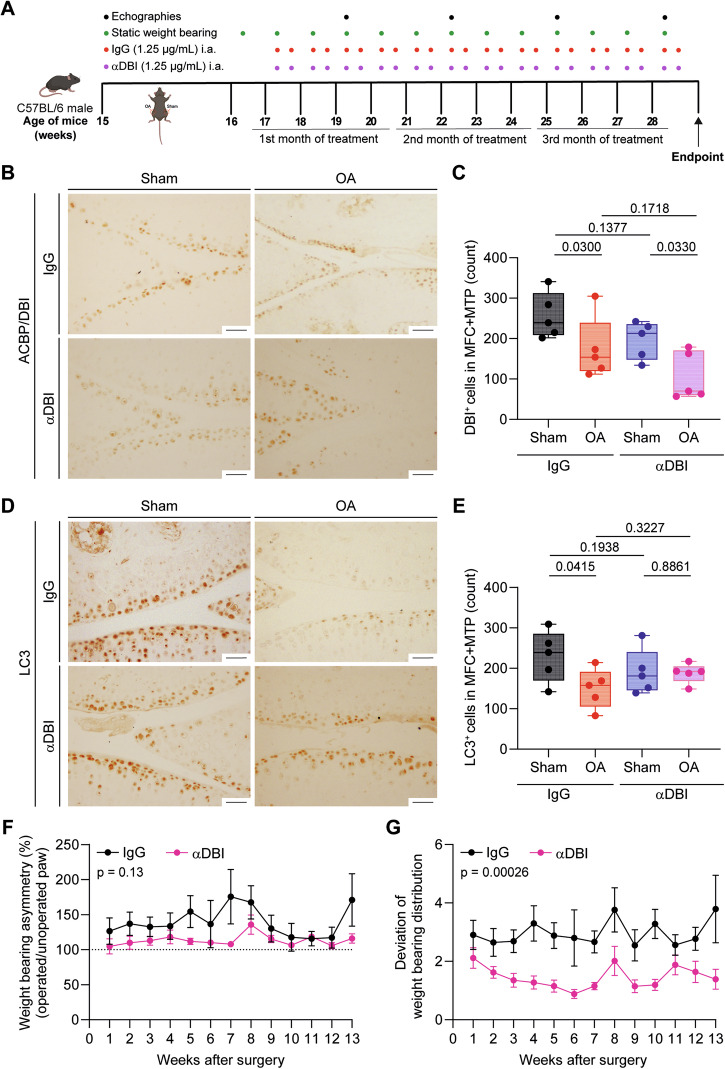
Effect of α-DBI intraarticular injections on cartilage integrity and weight-bearing asymmetry in OA Mice. **A** Schematic representation of the experimental timeline for intraarticular (i.a.) injections of α-DBI and preclinical monitoring in C57Bl/6 OA mice. In 15-week-old male C57Bl/6 mice, mechanical destabilization of the right knee joint was induced via MCL/DMM surgery, while the left knee underwent sham surgery. After a 2-week post-surgical recovery, i.a. injections of α-DBI or isotype control antibody were administered twice weekly for 12 weeks. To manage preclinical follow-up, postural balance measurement began at 16 weeks of age weekly and ultrasound scans were performed every 3 weeks. The mice were euthanized at 28 weeks of age. Schematic was generated using BioRender. Representative knee sections stained for ACBP/DBI (**B**) and LC3 (**D**). Scale bar, 50 μm. Quantification of ACBP/DBI-positive cells (**C**) and LC3-positive cells (**E**) in the medial femoral condyle (MFC) and medial tibial plateau (MTP). Results are displayed as box-and-whisker plots, with each dot representing an individual mouse (*n* = 5 mice per condition). For statistical analysis, *p* values were extracted from 2-way linear models, testing treatment significance within surgery status (formula: Count ~ Surgery/Treatment) and testing surgery significance within different treatments (formula: Count ~ Treatment/Surgery). Weight-bearing asymmetry (**F**) and the standard deviation of weight-bearing distribution (**G**) were evaluated over a 3-s period. Ten measurements were taken per mouse, and data are presented as the percentage ratio between weight-bearing on the right and left limbs (**F**) and the standard deviation of the ratio between the right and left knees (**G**). Mean ± SEM values (*n* = 10–13 mice per condition) are shown. Statistical analysis was performed using the area under the curve (AUC), and *p* values were calculated using ANOVA followed by Tukey’s test for multiple comparisons.

In the next series of experiments, we explored the possibility of treating OA locally, by intraarticular (i.a.) injections of α-DBI. The right knee joints of 15-week-old male C57Bl/6 mice were subjected to mechanical destabilization by MCL + DMM surgery, while the left knees underwent sham surgery. After a post-surgical recovery phase of 2 weeks, the affected joint was subjected to repeated i.a. injections of α-DBI (10 ng of antibody in 8 µL of vehicle) or isotype control antibody twice weekly for 12 weeks until 28 weeks of age and then were euthanized (Fig. 4A). At endpoint, α-DBI induced a reduction of immunohistochemically detectable DBI-positive cells in OA joints (Fig. 4B, C). We also observed that OA joints injected with isotype control antibody exhibited a reduction in autophagic (LC3B-positive) cells. This LC3B reduction was suppressed by α-DBI (Fig. 4D, E), indicating that local ACBP/DBI neutralization may restore normal levels of autophagy.

The combination of pain and mechanical joint failure resulting from OA causes mice to asymmetrically distribute their weight between their legs [41]. Quantitation of the asymmetry of weight distribution by means of a dynamic balance demonstrated that mice subjected to joint destabilization followed by i.a. injections of α-DBI exhibited less imbalance in their body weight distribution than control animals treated with the isotype control antibody (Fig. 4F, G).

These findings support the conclusion that treatment of OA by i.a. injections of α-DBI improves functional outcome. Encouraged by these findings, we performed additional sonographic, histological, and mechanistic studies of this local OA treatment.

### Time-dependent improvement of sonographic signs of OA by ACBP/DBI neutralization

**Fig. 5:**
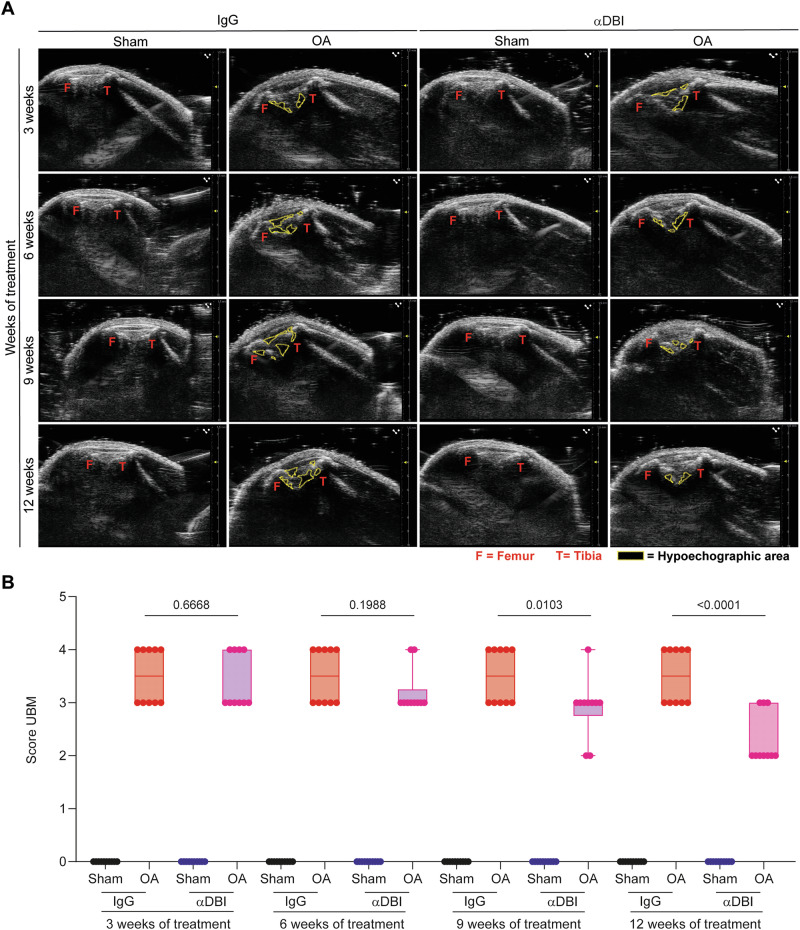
ACBP/DBI neutralization improves radiological signs of osteoarthritis, as determined by ultrasound biomicroscope B-mode. **A** Echographies of knees after 3,6,9,12 weeks of treatment with IgG and αACBP/DBI mAb clone 7G4a. Tibio-femoral triangle of sham and OA knees was taken using the ultrasound biomicroscope (UBM) in B mode. The femur and tibia are represented in red letters and hypoechogenic zones with a yellow dashed line. **B** Inflammation kinetic analysis according to the UBM score is shown. Results are displayed as box-and-whisker plots, with each dot representing an individual mouse (*n* = 9–10 mice per condition). For statistical analysis, *P* values were extracted from 2-way linear models, testing treatment significance within the week number of treatment (formula: Count ~ Week/Treatment).

We determined the evolution of OA by monthly non-invasive assessments of joint inflammation using a UBM. We observed that the areas occupied by the tibia-femur triangle, as well as hypoechogenic zones corresponding to inflamed tissue with a high content of fat, liquid, or semi-solid material [42, 43] expanded in OA knees as compared to sham-operated controls. Both these OA-associated alterations were mitigated by injection of anti-ACBP/DBI mAb clone 7G4a (the same used as above in Figs. 3 and 4) (Fig. 5).

As described previously [28], mAb 7G4a detects recombinant mouse (but not human) ACBP/DBI protein in immunoblots. A new mAb, 82B2G9 (dubbed 82), which was generated by hybridoma technology, recognized both human and mouse ACBP/DBI (Supplementary Fig. 4A). Of note, both mAbs recognize different epitopes in mouse ACBP/DBI, likely explaining their differential cross-reactivity with respect to human ACBP/DBI (Supplementary Fig. 4B, C). To evaluate the in vivo activity of both mAb, we selected the model of acetaminophen (APAP)-induced hepatotoxicity. This model provides a robust platform to assess the efficacy of ACBP/DBI inhibition [49]. This model offers clear and quantifiable endpoints, such as liver damage and necrosis, allowing us to rigorously evaluate the hepatoprotective effects of ACBP/DBI inhibition [26]. Importantly, i.p. injection of both antibodies similarly reduced acetaminophen (APAP)-induced hepatotoxicity (Supplementary Fig. 4D–F) as well as liver damage induced by bile duct ligation (Supplementary Fig. 4G–I), indicating that they effectively inhibit endogenous mouse ACBP/DBI in vivo. Accordingly, i.a. injected mAb 82B2G9 was as efficient as mAb 7G4a in mitigating OA in the mouse model (Supplementary Fig. 5). Notably, both antibodies (7G4a and 82B2G9) similarly reduced the UBM scores overtime with a maximum effect at the end of experiment (Fig. 5, Supplementary Fig. 5), suggesting that they have curative (rather than merely preventive) effects on OA. Hence, distinct anti-ACBP/DBI mAbs, including a cross-species-reactive mAb, can be used for treating OA.

### Mitigation of histological OA and systemic inflammation by intraarticular ACBP/DBI neutralization

**Fig. 6:**
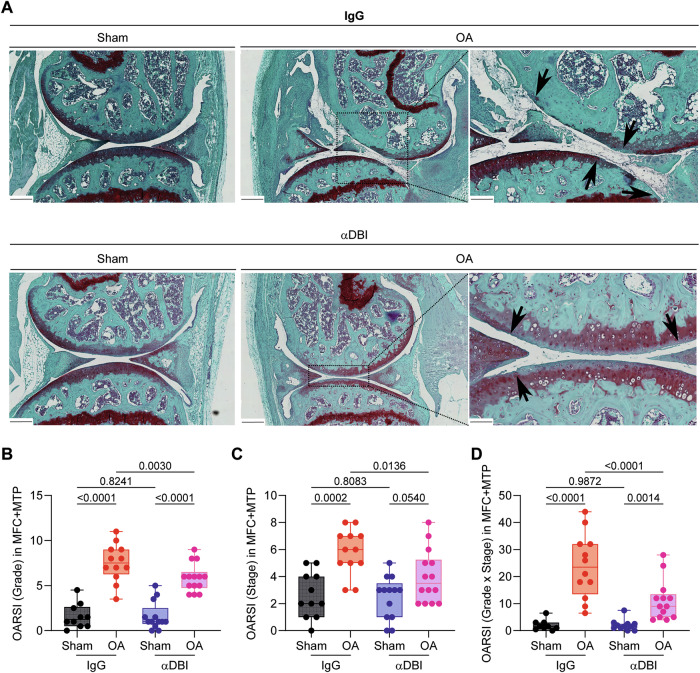
Intraarticular injection of anti-ACBP/DBI monoclonal antibody reduced cartilage destruction in osteoarthritis. **A** Representative Safranin O-Fast Green stained knee sections from mice treated with α-DBI or isotype control antibody. MCL/DMM causes erosion of calcified cartilage extending over 50% of the articular surface in the femur area. Furthermore, the damage starts to extend done the anterior part of the tibial area. In contrast, α-DBI attenuates cartilage loss. Black arrows indicate cartilage lesion. Scale bars 250 and 100 μm. **B** Semiquantitative scoring system about histological changes. The minimum value 0 corresponds to normal cartilage and the maximum value 12 represents the sum of the destroyed cartilage in MFC and MTP. **C** Represents the area occupied by the damage in the cartilage, where the value 0 is intact cartilage and the value 8 is the maximum, corresponding to the sum MFC + MTP. **D** Represents the relationship between the damage and the occupied area. The value 0 is the minimum and 48 is the maximum. Results are displayed as box-and-whisker plots, with each dot representing an individual mouse (*n* = 9–14 mice per condition). For statistical analysis, *p* values were extracted from 2-way linear models, testing treatment significance within surgery status (formula: Count ~ Surgery/Treatment) and testing surgery significance within different treatments (formula: Count ~ Treatment/Surgery).

**Fig. 7:**
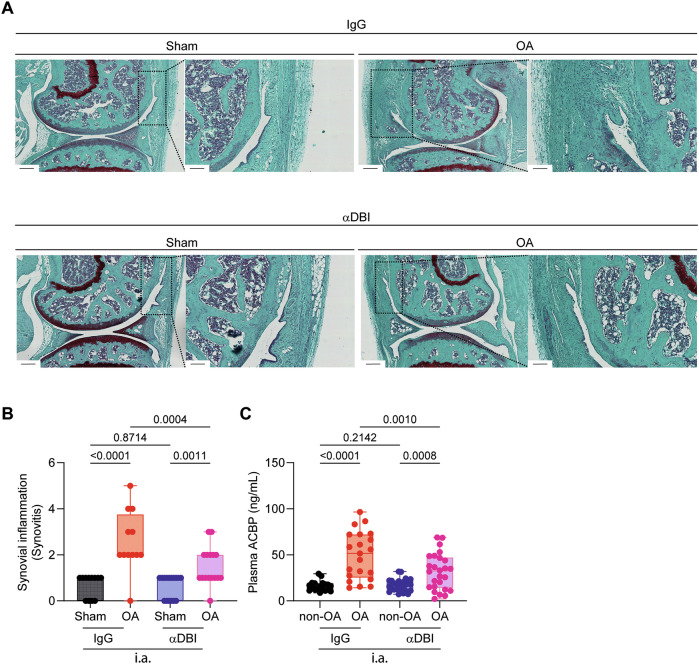
ACBP/DBI neutralization reduces synovial inflammation in osteoarthritis mice. **A** Representative images of knee synovial membrane from mice treated with α-DBI or isotype control antibody, stained with Safranin O. Scale bars 250 and 100 μm. **B** Quantification of Krenn score to determine synovitis. Data for each mouse range from 0 (no synovitis) to 9 (maximal inflammation) (*n* = 12–16 mice per group). For statistical analysis, *p* values were extracted from 2-way linear models, testing treatment significance within surgery status (formula: Count ~ Surgery/Treatment) and testing surgery significance within different treatments (formula: Count ~ Treatment/Surgery). **C** Plasma ACBP/DBI levels in Sham and OA mice treated by i.a. injections. Results are displayed as box-and-whisker plots, with each dot representing an individual mouse (*n* = 9–26 mice per condition). For statistical analysis, *p* values were calculated using two-way ANOVA corrected for multiple comparisons.

Histological analyses performed at the endpoint confirmed that ACBP/DBI neutralization significantly (*p* < 0.05, two-way ANOVA) reduced cartilage destruction, as quantified at the levels of OARSI grade, stage, and score, when compared to the isotype control antibody (Fig. 6A–D). In addition, anti-ACBP/DBI mAb caused a significant (*p* < 0.05, two-way ANOVA) diminution of synovial inflammation, as quantified by means of a standardized histopathological scoring system (Fig. 7A, B). Thus, local ACBP/DBI neutralization by i.a. injection of α-DBI mitigates histological signs of OA-associated synovitis.

**Fig. 8:**
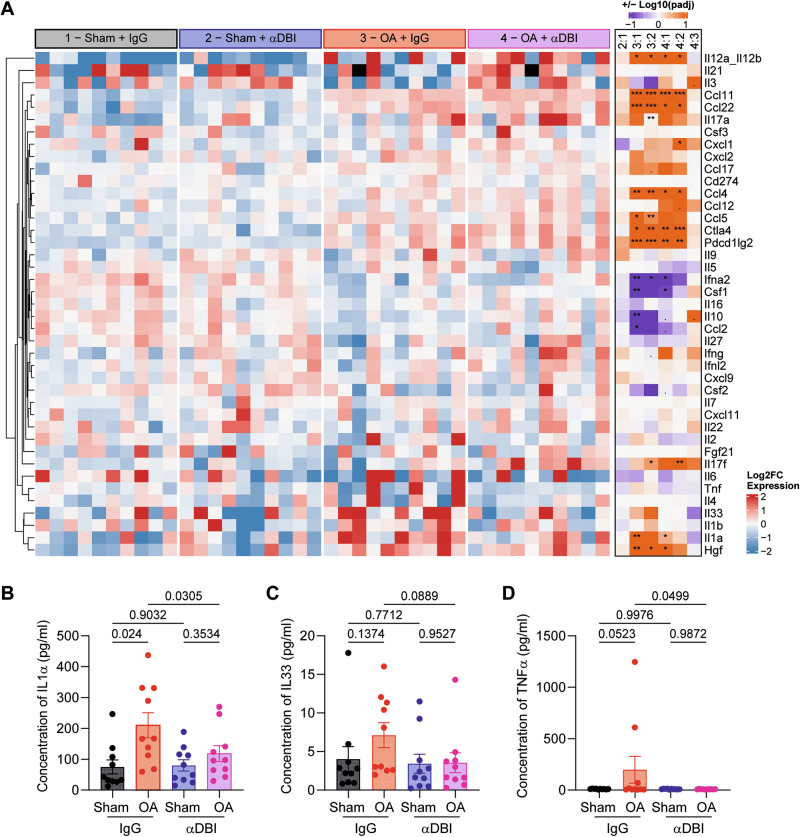
ACBP/DBI neutralization reduces pro-inflammatory cytokines in osteoarthritis mice. **A** Heatmap representation of the pro-inflammatory cytokine panel in mouse plasma treated with α-DBI or isotype control antibody. The normality of log2-transformed values was tested using the Shapiro–Wilk test. Cytokines with a normal distribution were analyzed by two-way ANOVA with Tukey’s HSD for pairwise comparisons. Non-normally distributed cytokines were analyzed using the Kruskal–Wallis test, followed by Dunn’s post-hoc test with Benjamini-–Hochberg correction for multiple comparisons. Individual representation of cytokine levels for (**B**) IL1α, (**C**) IL33, and (**D**) TNFα. Results are displayed as column plots, with each dot representing an individual mouse (*n* = 10 mice per condition), shown as mean ± SEM. For statistical analysis, *p* values were calculated using two-way ANOVA followed by Tukey’s test for multiple comparisons.

Additionally, the local (i.a.) injection of α-DBI reduced the OA-associated surge in systemic plasma ACBP/DBI concentrations (Fig. 7C), suggesting that the observed effect is primarily due to the local anti-inflammatory (and anti-OA) effects of α-DBI rather than to systemic spillover of the antibody. Consistent with this, we observed that in control mice receiving IgG control antibody, the induction of OA led to an increase in the plasma level of several pro-inflammatory cytokines (IL1α, IL33, TNFα) in the IgG control group that was abolished by i.a. α-DBI (Fig. 8A–D).

In conclusion, local inhibition of ACBP/DBI reduces both local and systemic signs of OA-associated inflammation.

## Discussion

In the present work, we demonstrate that local administration of anti-ACBP/DBI mAb convergently attenuated the histological, sonographic, functional, and inflammatory manifestations of OA. We conclude that inhibition of ACBP/DBI by means of neutralizing mAbs injected into affected joints has therapeutic effects against OA.

Thus far, we have not investigated optimal dosing and scheduling of intraarticular anti-ACBP/DBI mAb. In our study, we injected 10 ng of ACBP/DBI mAbs twice per week (amounting to a total of 20 ng per week) through the intraarticular route. This is less than the dose of ACBP/DBI required for protecting the heart, liver, or lung against damage. Indeed, for cardioprotection (against infarction or anthracycline toxicity), hepatoprotection (against acetaminophen, bile duct ligation, carbon tetrachloride, ischemia-reperfusion or dietary insults), or pneumoprotection (against bleomycin-induced inflammation and fibrosis), we injected higher doses of ACBP/DBI, usually 2.5–5 µg per g body weight per week via the intraperitoneal route, which for a mouse with a body weight of 20 g would correspond to 50–100 µg of anti-ACBP/DBI mAb per week [26]. Since antibodies injected into the synovial cleft are not as free to diffuse into other tissues (although some leakage can occur) [50, 51] as antibodies injected into the peritoneal cavity, it is conceivable that intraarticular administration of ACBP/DBI mAb could be effectuated at even lower doses and/or at lower frequencies to obtain therapeutic effects. In this context, it may be important to study the pharmacokinetics of intraarticularly injected ACBP/DBI mAb. However, thus far we have not observed any long-term toxicities of systemically (intraperitoneally) injected anti-ACBP/DBI antibodies. Similarly, autovaccination with ACBP/DBI aimed at inducing neutralizing antibodies, has shown no adverse effects. Additionally, permanent genetic modification of the ACBP/DBI system (by inducible knockout of the *Dbi* gene or constitutive point mutation *Gabrg2*^F77I/F77I^ of the ACBP/DBI receptor) has also been compatible with the maintenance of a healthy state in long-term experiments [26, 27, 31, 52, 53]. Hence, the possible leakage of intraarticularly injected ACBP/DBI antibody cannot be considered as a health hazard. Accordingly, systemic neutralization of ACBP/DBI, for instance by subcutaneous depot injections, might constitute an alternative to its i.a. administration.

In our previous publications on ACBP/DBI, we have used one single antibody, 7G4a, which is a mAb obtained by conventional hybridoma technology after mice were vaccinated with keyhole limpet cyanine-conjugated mouse ACBP/DBI protein through a procedure that breaks self-tolerance [54]. 7G4a mAb was found to mediate broad organ-protective effects in mice, and these effects could be mimicked by knockout of *Dbi* or *Gabrg2*^F77I/F77I^ mutation, strongly suggesting that 7G4a acts on target, via the neutralization of ACBP/DBI and not that of other factors [26]. Accordingly, the anti-OA effects of 7G4a resembled those of the knockout of *Dbi*, supporting the idea that 7G4a acts on target. As shown here, 7G4a mAb recognizes mouse, not human ACBP/DBI protein. In contrast, a new mAb, 82B2G9 (dubbed 82) is an interspecies crossreactive mAb recognizing both human and mouse ACBP/DBI could be generated. This mAb mimicked the effects of 7G4a with respect to OA mitigation. mAb 82B2G9 bears the framework and constant regions of a mouse IgG1 antibody. However, it is theoretically possible to graft the complementarity-determining regions of this mouse antibody on a human backbone (de facto replacing the mouse-specific portions by the human framework and constant regions), hence obtaining the “humanization” of this mAb, which would continue to recognize human ACBP/DBI protein. From this point of view, mAb 82B2G9 (or other mouse/human ACBP/DBI-crossreactive antibodies) might constitute a starting point for the clinical development of therapeutic ACBP/DBI antibodies.

Mechanistically, anti-ACBP/DBI mAb falls into the category of autophagy inducers [26], commensurate with current evidence that OA can be prevented or treated by the stimulation of autophagy [13, 55–57], which inhibits apoptotic cell loss in OA [12, 56, 58]. It should be noted that preclinical models of OA mainly utilize male mice due to their capacity to manifest fast-progressing OA, a phenotype that is only observed in an attenuated fashion in female mice [59]. Admittedly, this specificity of mice does not reflect the human pathology, in which knee OA is more prevalent in females than in males and hip OA is more prevalent in females if it is clinically or symptomatically defined (but more prevalent in males if it is defined by radiographic criteria) [60]. In any case, the late and attenuated development of OA in female mice renders the utilization of mouse dams impractical for the study of OA therapies. However, the role of ACBP/DBI in various diseases is not sex-specific since both male and female mice respond to recombinant ACBP/DBI administration, as well as to the injection of the anti-ACBP/DBI antibody, in mouse models of anorexia [61], Cushing syndrome [62] and steatohepatitis [26].

It is important to note that induction of OA in mice led to an increase in plasma ACBP/DBI concentrations. Conversely, local injection of anti-ACBP/DBI antibody blunted the systemic increase of ACBP/DBI induced by OA. Since i.a. anti-ACBP/DBI antibody injections did not reduce circulating ACBP/DBI concentrations measurable by ELISA in control mice without OA, it appears that the effects of the anti-ACBP/DBI antibody on the OA-associated surge in plasma ACBP/DBI are secondary to its local anti-inflammatory effects. At this point, it is not clear whether the increase of ACBP/DBI observed in untreated OA can be attributed to locally produced ACBP/DBI protein or whether it reflects a systemic response to local injury. To solve this question, a series of cell type-specific *Dbi* knockouts (e.g., in myeloid or cartilage cells) must be performed in the future. Similarly, the tissue of origin of two alarmins, IL1α and IL33 (which are released from the nuclei of injured cells) [63] and that of TNFα (which is actively secreted by immunocytes) [64] remains to be determined. In the OA mouse model, plasma IL1α, IL33, and TNFα increased, and this effect was suppressed by i.a. injections of α-DBI. Clinical studies using neutralizing reagents against IL1α or TNFα have failed [65–67], suggesting that these pro-inflammatory factors do not constitute useful therapeutic targets for the treatment of OA. However, together with ACBP/DBI, they might be explored as biomarkers of OA progression and therapeutic responses. Finally, it is important to note that IL1β, IL6 and TNFα upregulate ACBP/DBI and GABRG2 protein expression in human chondrocyte and synoviocyte cells, supporting the widespread idea that many different cell types contribute to joint inflammation in osteoarthritis together with changes in the extracellular matrix [18, 68–70]. We are currently elaborating a single-cell expression atlas to identify the multiple sources of ACBP/DBI [71]. It will be important to confront such single-cell data regarding GABRG2, cytokines, and cytokine receptors to launch bioinformatic simulations of the pro-inflammatory cascade that determines OA pathogenesis.

In conclusion, local injection of anti-ACBP/DBI mAbs has therapeutic activity against OA, a condition that constitutes a major unmet medical need. This approach offers a novel and promising strategy to halt OA progression and preserve joint function. It will be worthwhile to follow up these initial observations in rodents with experiments on larger animals that more closely mimic human (patho)physiology to fully evaluate the translational potential of this autophagy-inducing strategy for OA therapy.

## Supplementary information

Raw_Data_WB_Supplementary Material

Supplementary Material

## Data Availability

The data that support the findings of this study are available from the corresponding authors upon reasonable request.
