# Supplementary material for: Neutralization of acyl CoA binding protein (ACBP) for the experimental treatment of osteoarthritis
Source: Cell Death Differ. 2025 Mar 13;32(8):1484–98. doi: 10.1038/s41418-025-01474-y (PMC12326017; doi:10.1038/s41418-025-01474-y)

Experiment 1: TC28a2_24h

GABA

Marker

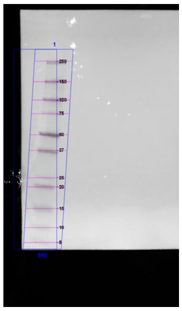

GABA Antibody

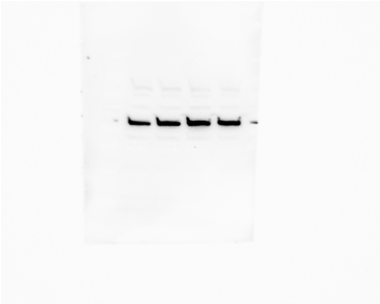

Marker+GABA

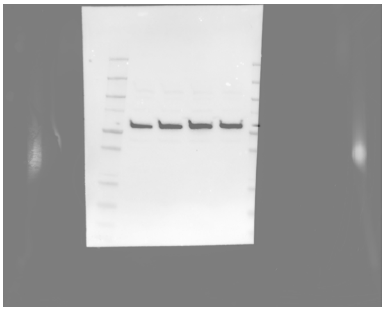

Volume of GABA – 3D Image

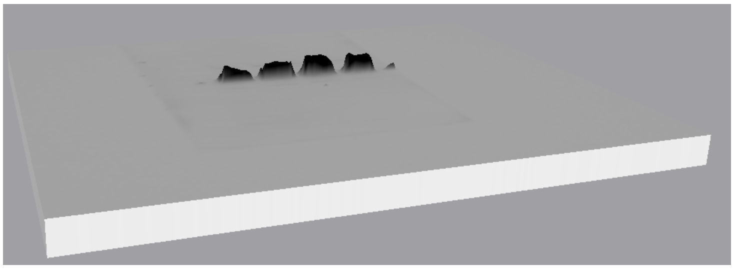

DBI

Marker

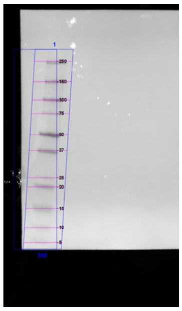

DBI Antibody

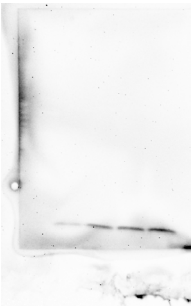

Marker+DBI

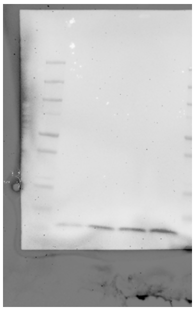

Volume of DBI – 3D Image

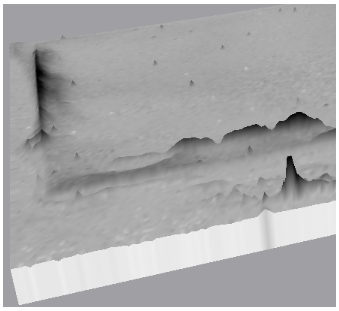

Experiment 1: TC28a2_24h

GAPDH

Marker

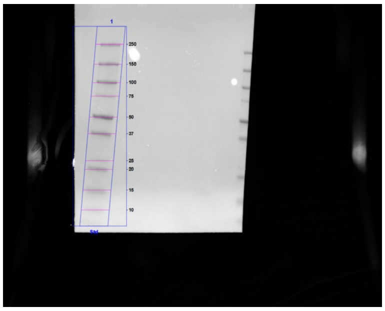

GAPDH Antibody

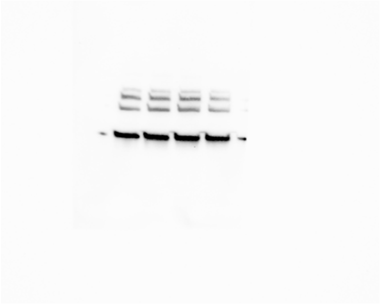

Marker+GAPDH

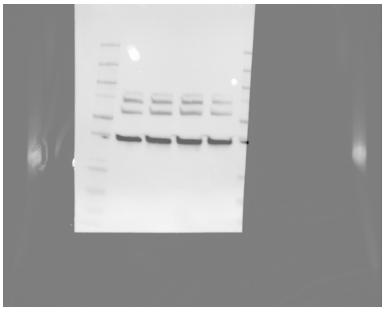

Volume of GAPDH – 3D Image

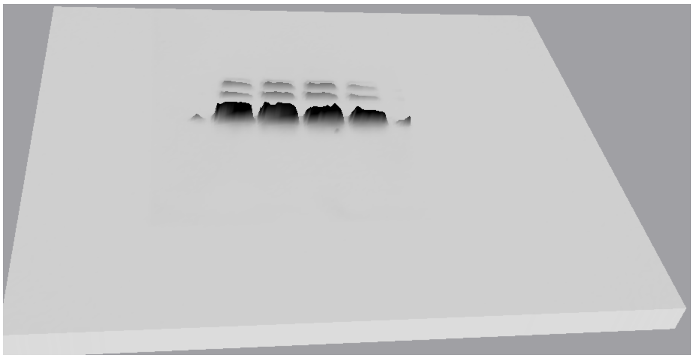

Experiment 2: TC28a2_24h

GABA

Marker

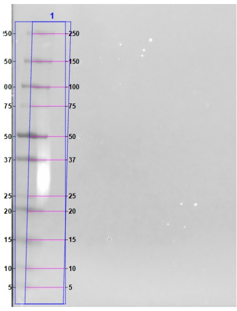

GABA Antibody

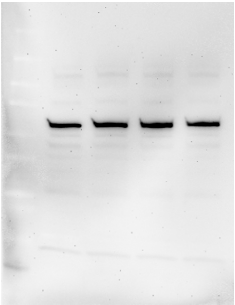

Marker+GABA

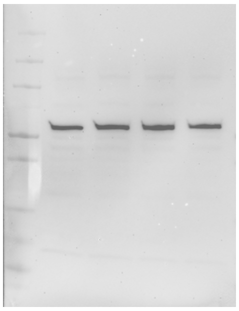

Volume of GABA – 3D Image

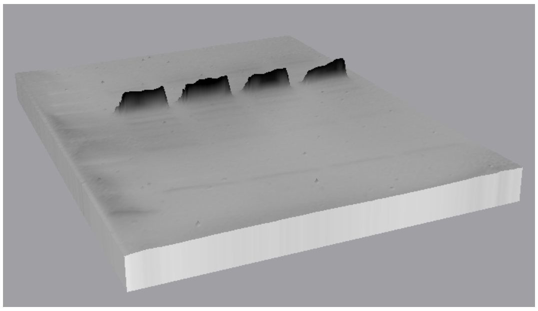

DBI

Marker

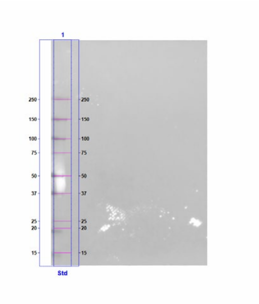

DBI Antibody

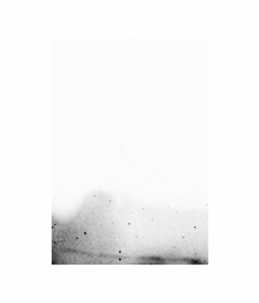

Marker+DBI

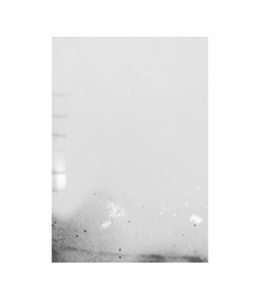

Volume of DBI – 3D Image

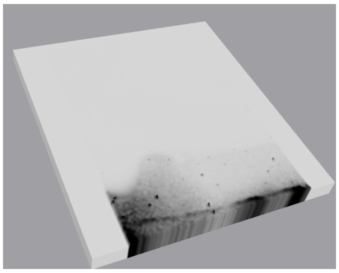

Experiment 2: TC28a2_24h

GAPDH

Marker

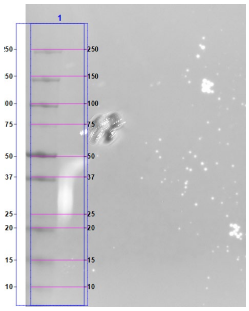

GAPDH Antibody

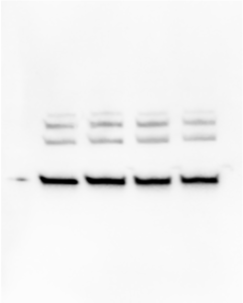

Marker+GAPDH

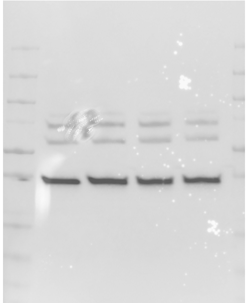

Volume of GAPDH – 3D Image

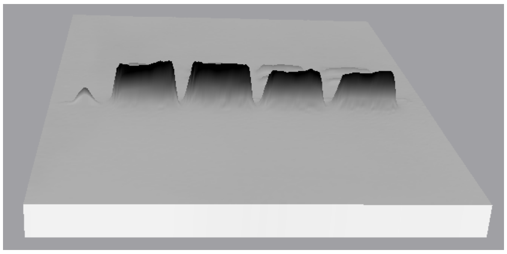

Experiment 3: TC28a2_24h

GABA

Marker

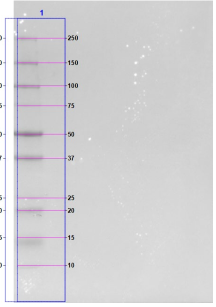

GABA Antibody

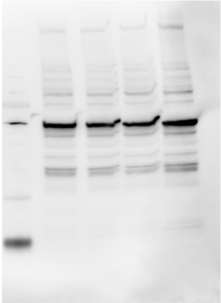

Marker+GABA

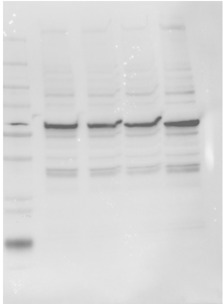

Volume of GABA – 3D Image

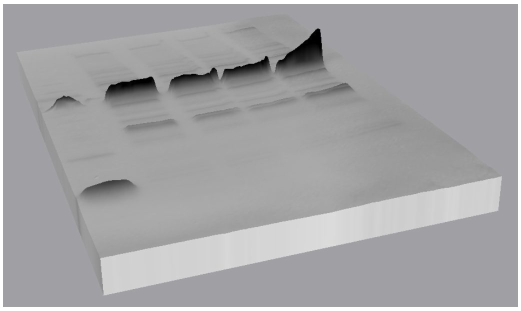

DBI

Marker

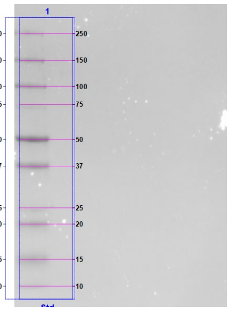

DBI Antibody

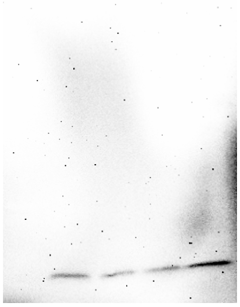

Marker+DBI

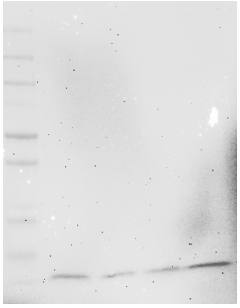

Volume of DBI – 3D Image

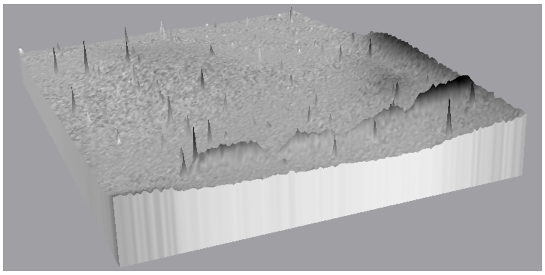

Experiment 3: TC28a2_24h

GAPDH

Marker

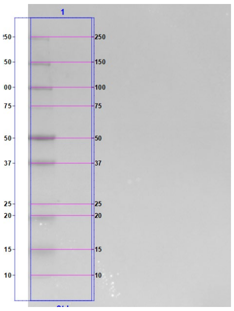

GAPDH Antibody

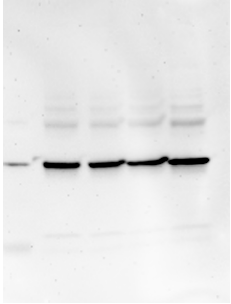

Marker+GAPDH

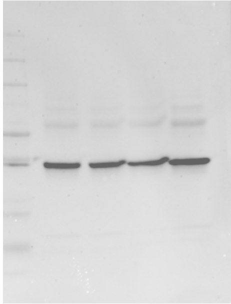

Volume of GAPDH – 3D Image

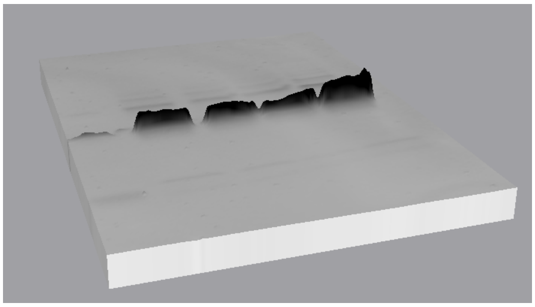

Experiment 1: SW982_48h

GABA

Marker

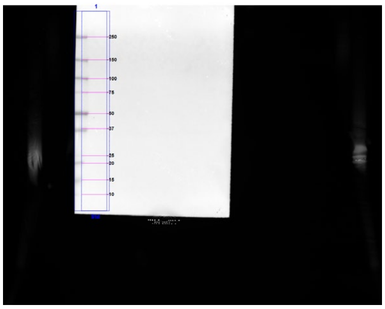

GABA Antibody

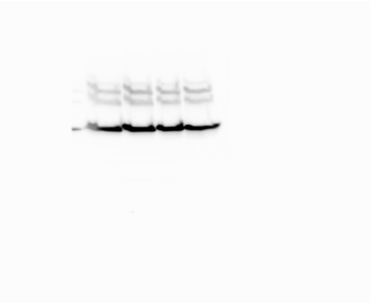

Marker+GABA

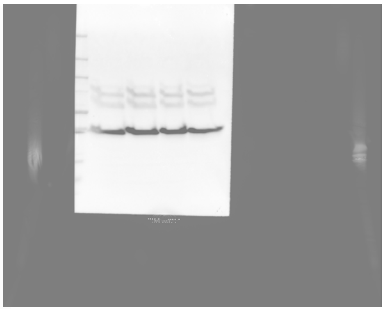

Volume of GABA – 3D Image

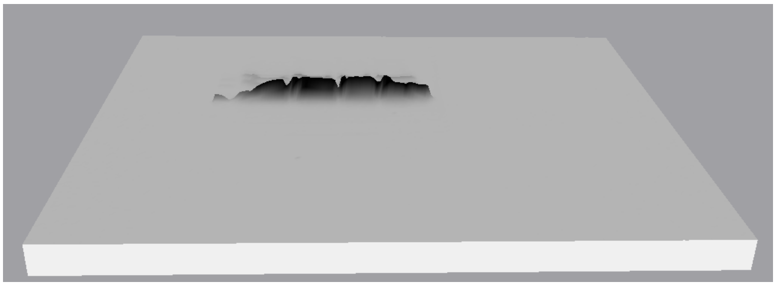

DBI

Marker

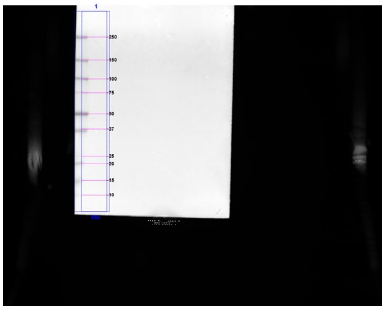

DBI Antibody

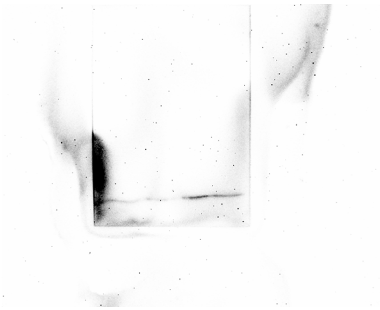

Marker+DBI

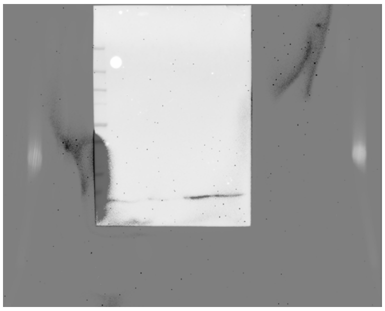

Volume of DBI – 3D Image

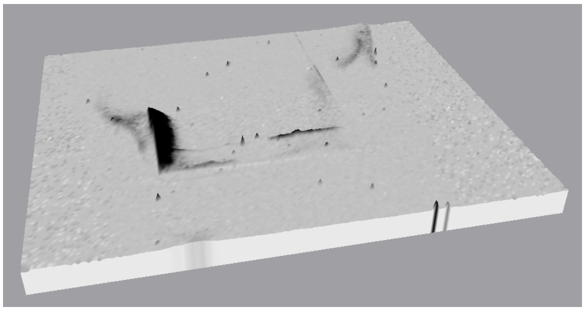

Experiment 1: SW982_48h

GAPDH

Marker

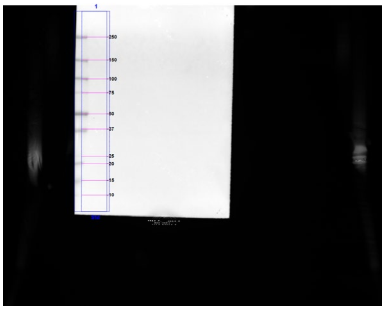

GAPDH Antibody

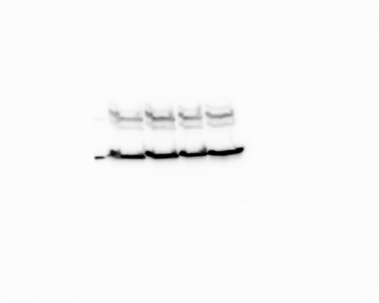

Marker+GAPDH

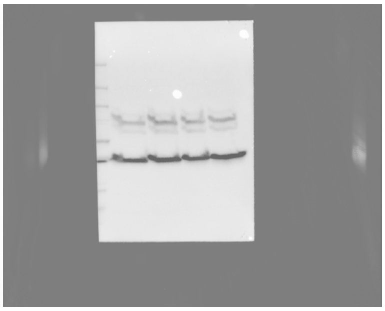

Volume of GAPDH – 3D Image

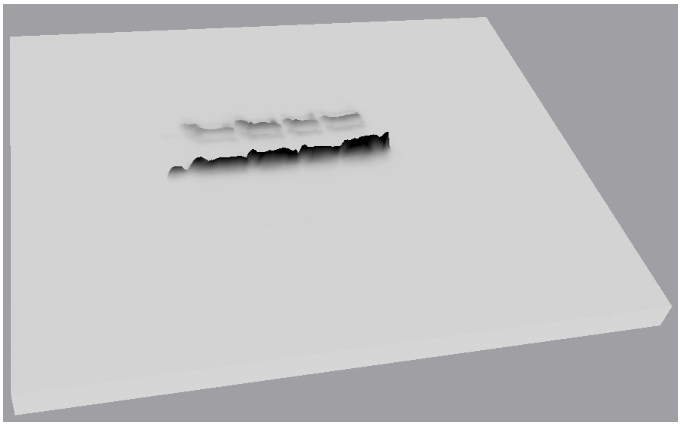

Experiment 2: SW982_48h

GABA

Marker

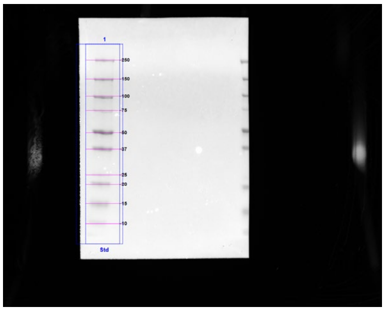

GABA Antibody

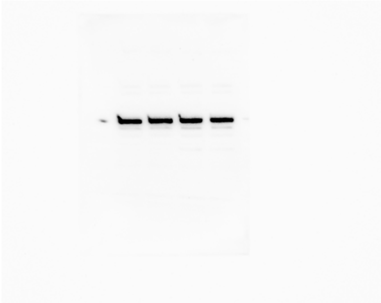

Marker+GABA

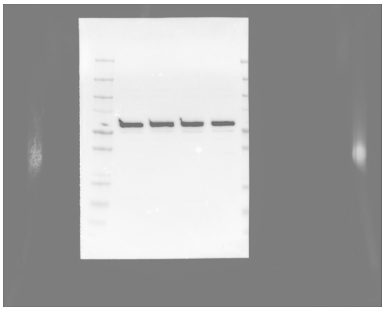

Volume of GABA – 3D Image

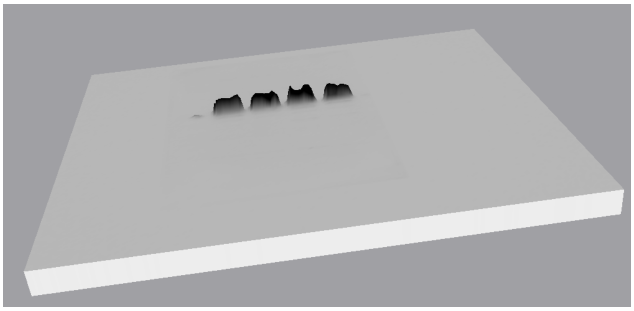

DBI

Marker

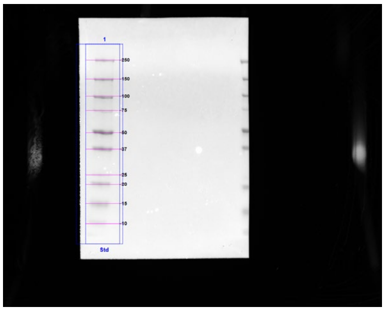

DBI Antibody

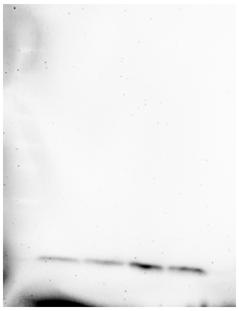

Marker+DBI

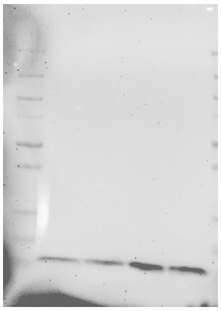

Volume of DBI – 3D Image

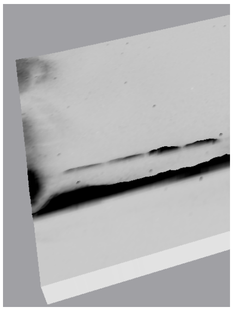

Experiment 2: SW982_48h

GAPDH

Marker

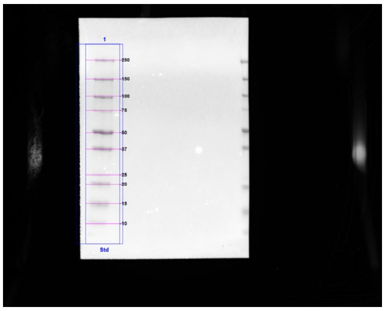

GAPDH Antibody

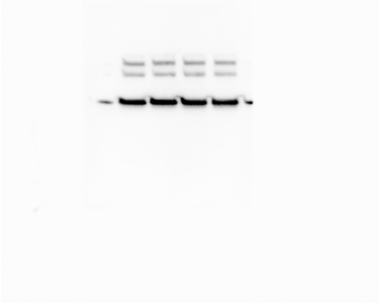

Marker+GAPDH

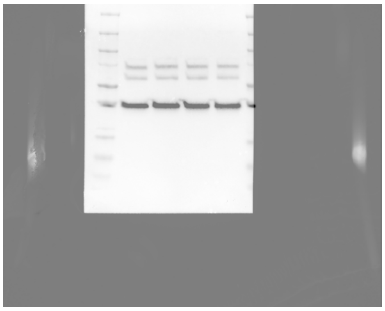

Volume of GAPDH – 3D Image

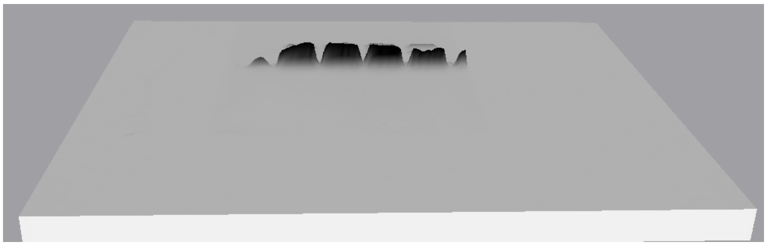

Experiment 3: SW982_48h

GABA

Marker

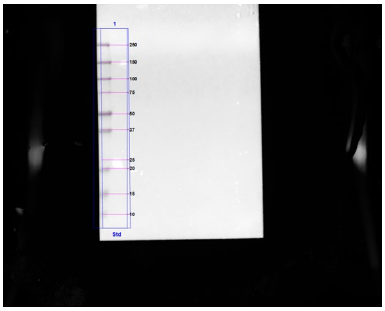

GABA Antibody

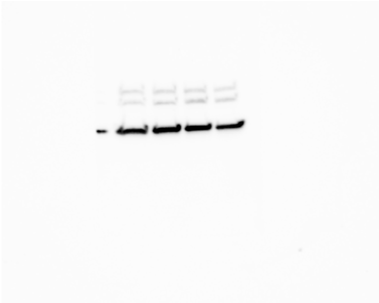

Marker+GABA

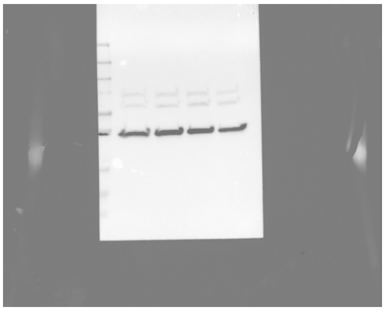

Volume of GABA – 3D Image

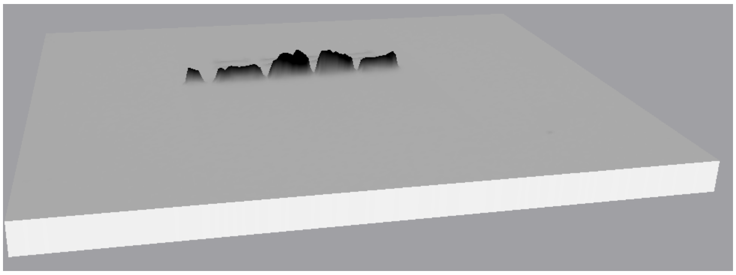

DBI

Marker

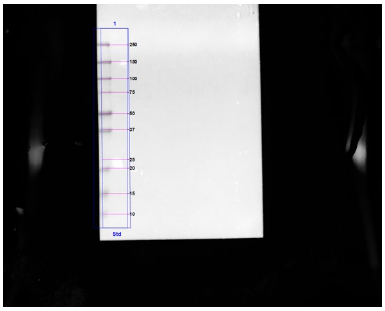

DBI Antibody

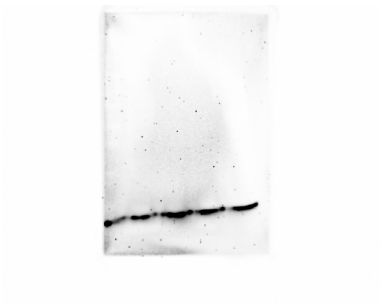

Marker+DBI

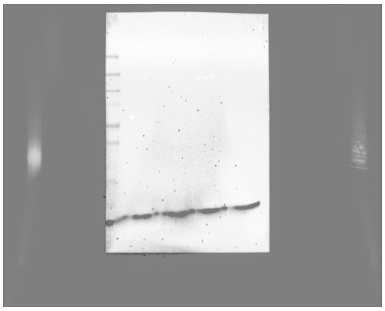

Volume of DBI – 3D Image

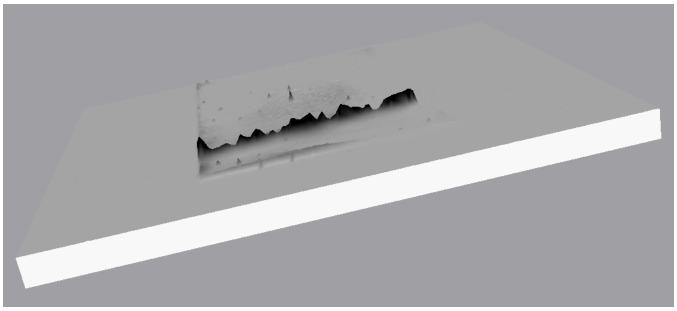

Experiment 3: SW982_48h

GAPDH

Marker

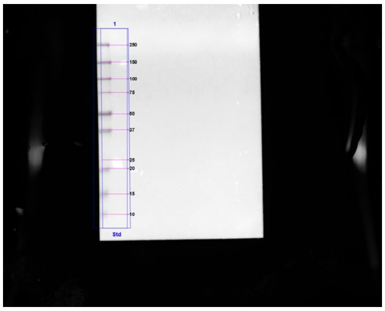

GAPDH Antibody

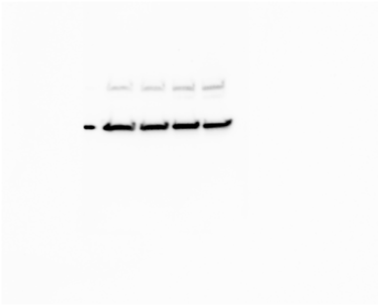

Marker+GAPDH

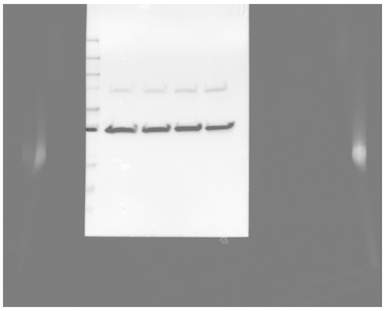

Volume of GAPDH – 3D Image

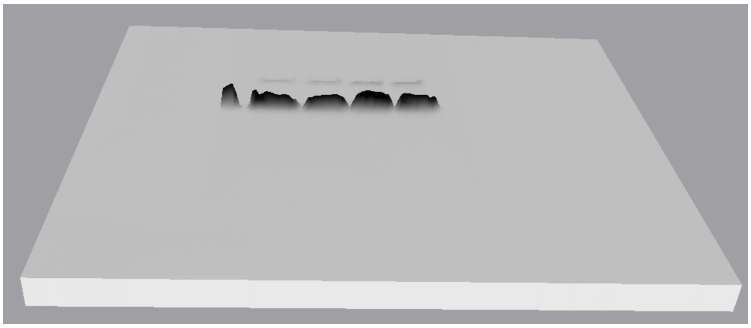

Supplement: Supplementary file 1 — Raw_Data_WB_Supplementary Material [file 41418_2025_1474_MOESM1_ESM.pdf]
