# Supplementary material for: Neutralization of acyl CoA binding protein (ACBP) for the experimental treatment of osteoarthritis
Source: Cell Death Differ. 2025 Mar 13;32(8):1484–98. doi: 10.1038/s41418-025-01474-y (PMC12326017; doi:10.1038/s41418-025-01474-y)
Supplement: Supplementary file 2 — Supplementary Material [file 41418_2025_1474_MOESM2_ESM.docx]

**Neutralization of acyl CoA binding protein (ACBP) for the experimental treatment of osteoarthritis**

**Uxía Nogueira-Recalde^1,2,3^, Flavia Lambertucci^1,2^, Léa Montégut^1,2^, Omar Motiño^1,2,4^, Hui Chen^1,2^, Sylvie Lachkar^1,2^, Gerasimos Anagnostopoulos^1,2^, Gautier Stoll^1,2^, Sijing Li^1,2^, Vincent Carbonier^1,2^, Ester Saavedra Díaz^5^, Francisco J Blanco^3^, Geert van Tetering^6^, Mark de Boer^6^, Maria Chiara Maiuri^1,2,7^, Beatriz Caramés^3^, Isabelle Martins^1,2^, Guido Kroemer^1,2,8^**

^1^ Centre de Recherche des Cordeliers, Equipe Labellisée par la Ligue Contre le Cancer, Université de Paris Cité, Sorbonne Université, Paris, France ;

^2^ Metabolomics and Cell Biology Platforms, Gustave Roussy Cancer Center, Villejuif, France

^3^ Unidad de Biología del Cartílago, Grupo de Investigación en Reumatología (GIR), Instituto de Investigación Biomédica de A Coruña (INIBIC), Complejo Hospitalario Universitario de A Coruña (CHUAC), Sergas, Universidad de A Coruña (UDC), A Coruña, Spain;

^4^ Unidad de Excelencia, Instituto de Biología y Genética Molecular (IBGM), Universidad de Valladolid – CSIC, Valladolid, Spain;

^5^ Departamento de Bioquímica y Biología Molecular, Fisiología, Genética e Inmunología, Instituto Universitario de Investigaciones Biomédicas y Sanitarias (IUIBS), Universidad de Las Palmas de Gran Canaria, Las Palmas de Gran Canaria, Spain;

^6^ Osasuna Therapeutics, Lausanne, Switzerland;

^7^ Department of Molecular Medicine and Medical Biotechnologies, University of Napoli Federico II, Napoli, Italy;

^8^ Department of Biology, Institut du Cancer Paris CARPEM, Hôpital Européen Georges Pompidou, AP-HP, Paris, France.

Correspondence to	Guido Kroemer (kroemer@orange.fr) or

Isabelle Martins (isabelle.martins@inserm.fr)

**Supplemental Figures**

**Supplemental Figure 1.** ACBP/DBI and GABRG2 are upregulated in chondrocytes and synoviocytes exposed to inflammatory cytokines. Human T/C28a2 chondrocyte (A,B) and SW982 synoviocytes (C,D) were cultured in the presence of IL6 (20ng/ml), IL-1beta (5ng(ml) or TNF-alpha (10ng/ml) and then subjected to immunoblot detection of ACBP/DBI, GABRG2 and GAPDH. Representative immunoblots (1 out of 3 are shown in A,C) and quantitative results (B,D) are shown. Results are displayed as column plots (with each dot representing one quantification). Statistical analyses (*p* values) were calculated by means of the Kruskal-Wallis test with Benjamini, Krieger and Yekutieli correction.

**Supplemental Figure 2.** ACBP/DBI is expressed in human cartilage. (**A**) Representative images of healthy cartilage explants (Mankin score 0) and OA cartilage explants (Mankin score 8 and 12) stained with Safranin O-Fast Green and hACBP/DBI. Scale bar: 100 μm. Mean ± SD: 71.3 ± 23.07, mankin score 0, n = 3; mean ± SD: 80.33 ± 7.51, mankin score 8, n = 3; mean ± SD: 74 ± 9, mankin score 12, n = 3.

**Supplemental Figure 3.** GABRG2 is upregulated in OA human cartilage. (A) Human normal and OA cartilage was analyzed by immunohistochemistry for GABRG2. Representative images of healthy an OA cartilage stained with Safranin O-Fast Green and GABRG2. Scale bar, 100 μm and 50 μm. (**B**) Quantitative analysis of GABRG2-positive cells. Values are the mean ± SEM of n =5, Mankin score = 0, mean ± SD: 76.2 ± 18.42; n = 3, Mankin score = 8, mean ± SD: 89.33 ± 0.57; n = 4, Mankin score = 12, mean ± SD: 77.75 ± 10.5. For statistical analysis, P-values were extracted from a post-hoc pairwise test, regarding genotype significance.

**Supplemental Figure 4: Characterization of monoclonal antibodies against ACBP/DBI.** (**A**) Representative ACBP immunoblot from human and mouse recACBP/DBI (10, 25, 50, 100 ng) using the 82B2G9 antibody. (**B**-**C**). Depiction of putative epitopes onto a modeled structure for mouse ACBP. The model for the mouse ACBP was obtained from AlphaFold. The epitopes for 82B2G9 (**B**) and 7G4a (**C**) were highlighted by color. (**D**) Experimental procedure of the damage induced by acetaminophen (APAP, i.p. 300 mg/kg for 16 hours) in mice pre-treated with i.p. injection of α-ACBP/DBI or IgG (2.5 µg/g) for 4 hours and just before hepatic injury. ALT and AST transaminases activity are shown in (**E**) and (**F**) from plasma mice (n=5-16 mice per group). (G) Experimental procedure of liver damage induced by bile duct ligation (BDL) in mice pre-treated with i.p. injection of α-ACBP/DBI or IgG (2.5 µg/g) for 4 hours and 1 hour before hepatic injury. ALT and AST transaminases activity are shown in (H) and (I) from plasma mice (n=5-16 mice per group). Results are displayed as box-and-whisker plots (with each dot representing one single mouse). For statistical analysis, p-values were calculated using one-way ANOVA corrected for multiple comparisons. Schematics were generated using BioRender.

**Supplemental Figure 5.** **ACBP/DBI neutralization improves inflammation signs of osteoarthritis, as determined by ultrasound scan.** (**A**) Kinetic ultrasound representation of the tibiofemoral triangle of OA knees using the ultrasound biomicroscope (UBM) in B mode. Mice were treated with mAb 82B2G9 and IgG intraarticularly. Inflammation in the joint capsule was assessed every three weeks until 12 weeks of treatment. Ultrasound images of sham and OA knees were taken. The femur and tibia are represented in red letters and hypoechogenic areas with a yellow dashed line. (**B**) Inflammation analysis according to the UBM score. Results are displayed as box-and-whisker plots, with each dot representing an individual mouse (n = 6 to 10 mice per condition). For statistical analysis, p-values were extracted from 2-way linear models, testing treatment significance within different genotypes (formula: Count ~ Genotype/Treatment) and testing genotype significance within different treatments (formula: Count ~ Treatment/Genotype).
